# Porcine parvovirus infection activates mitochondria-mediated apoptotic signaling pathway by inducing ROS accumulation

**DOI:** 10.1186/s12985-016-0480-z

**Published:** 2016-02-16

**Authors:** Xiaomin Zhao, Hailing Xiang, Xiaoyuan Bai, Naijiao Fei, Yong Huang, Xiangjun Song, Hongling Zhang, Liang Zhang, Dewen Tong

**Affiliations:** College of Veterinary Medicine, Northwest A&F University, Yangling, Shaanxi 712100 People’s Republic of China

**Keywords:** Porcine parvovirus, Apoptosis, Mitochondria, Swine testicular cells, Reactive oxygen species

## Abstract

**Background:**

Porcine parvovirus (PPV) infection primarily causes reproductive failure of pregnant swine and results in host cell death. Boars, as an important disseminator, shed PPV to sows via semen. PPV infects and numerously replicates in boar testicle, which results in damage of swine testicle in vivo. Reactive oxygen species (ROS), a mediator of cell apoptosis, play a crucial role in the mitochondria apoptotic pathway. However, whether PPV infection induces ST cells apoptosis and ROS accumulation is still unclear.

**Methods:**

To determine the effects of PPV infection on the apoptosis, we detected morphological changes, DNA ladder, activities of caspases, and expression of PARP in PPV-infected ST cells. Moreover, aiming to investigate the effect of PPV infection on the mitochondrial apoptotic pathway and ROS accumulation, we detected the Δψm, apoptosis-related genes, and ROS. To investigate the role of ROS in the process of PPV-induced apoptosis, the ST cells were infected with PPV and treated with the ROS antioxidants. The ROS level was measured using Reactive Oxygen Species Assay Kit and the Δψm, expression level of Bcl-2, translocation of Bax, and redistribution of mitochondria cytochrome c were tested.

**Results:**

In this study, we demonstrated that PPV infection could induce apoptosis that was characterized by morphological changes, DNA fragmentation and activation of caspases. Moreover, PPV infection suppressed Bcl-2 expression, enhanced Bax expression and translocation to mitochondria, decreased the mitochondrial transmembrane potential, and triggered the release of cytochrome c, which caused the subsequent activation of caspase-9 and caspase-3 and initiation of apoptosis. However, during the process of PPV-induced apoptosis, the protein levels of Fas and FasL were not affected. Further studies showed that PPV infection caused ROS accumulation. Inhibition of ROS could reduce mitochondrial transmembrane potential and could significantly block ST cells apoptosis via suppressing Bax translocation, cytochrome c and caspase-3 activation.

**Conclusions:**

All these results suggest that PPV-induced ROS accumulation mediates apoptosis in ST cells, which provided theoretical basis for the molecular pathogenesis of PPV infection.

## Background

PPV is a member of parvoviridae, containing a negative single-stranded DNA genome [1, 2]. PPV infection may lead to reproductive failure, including infertility, embryonic death, stillbirth and fetal mummification in pregnant sows [3]. PPV infection mainly causes reproductive clinical syndrome, including infertility, abortion, stillbirth, neonatal death, and reduced neonatal vitality [4]. Boars play an important role in dissemination of PPV. It was reported that PPV infection damages both uterine [5] and testicles [6, 7] in vivo. Swine testicular is the reproductive organ and responsible for generating semen, which serve as one of the transmission routes of PPV. Therefore, we use ST cells to investigate the mechanism of PPV infection in this study.

The infection of animal virus such as human parvovirus [8, 9], canine parvovirus [10] may induce host cells apoptosis and is a major factor of cell death and tissue damage in viral diseases. Previous studies indicated that PPV infection caused apoptosis via p53 pathway in PK-15 cells [11]. Although, we previously demonstrated that PPV induced apoptosis PK-15 cells, whether PPV induces ST cells apoptosis and the role of ROS in apoptosis are unknown.

Apoptosis, a programmed cell death (PCD), is an important pathological cellular response. In vertebrate cells, the apoptotic pathways mainly include the extrinsic and the intrinsic pathways [12]. The extrinsic apoptotic pathway is induced by stimulation of death receptors that belongs to the tumor necrosis factor receptor (TNFR) family, such as Fas. The induction of intrinsic apoptotic pathway is associated with mitochondria upon activation that is highly regulated by ROS [13]. ROS, known as toxic products of cellular metabolism, are mainly produced by the mitochondria in most vertebrate cells and act as signaling molecules. Moreover, growing evidence suggests ROS are involved in regulation of many pathological processes such as cellular apoptosis [14, 15]. However excessive ROS is toxic to cells, especially in the process of apoptosis. It is indicated that ROS accumulation was able to enhance apoptosis by collapsing the mitochondrial potential, inducing mitochondrial oxidation channel, and releasing cytochrome C from mitochondria to the cytosol [16]. ROS accumulation is also linked to virus-induced apoptosis. For instance, parvovirus H-1 infection induces apoptosis via mediating ROS accumulation [17, 18]. We have reported that PPV infection could be able to trigger cell apoptosis through mitochondria-mediated pathway [19]. However, whether ROS are associated with PPV-induced apoptosis is still unclear.

In this study, we asked whether PPV infection could induce apoptosis via ROS accumulation in ST cells. To address this issue, we detected whether PPV infection causes apoptosis in ST cells. In addition, the ROS induced by PPV infection was assessed compared with mock infection. The results showed that PPV infection could triggers ST cell apoptosis via PPV-induced ROS accumulation.

## Results

### PPV infection induced apoptosis in ST cells through activating caspase-3 and -9

**Fig. 1:**
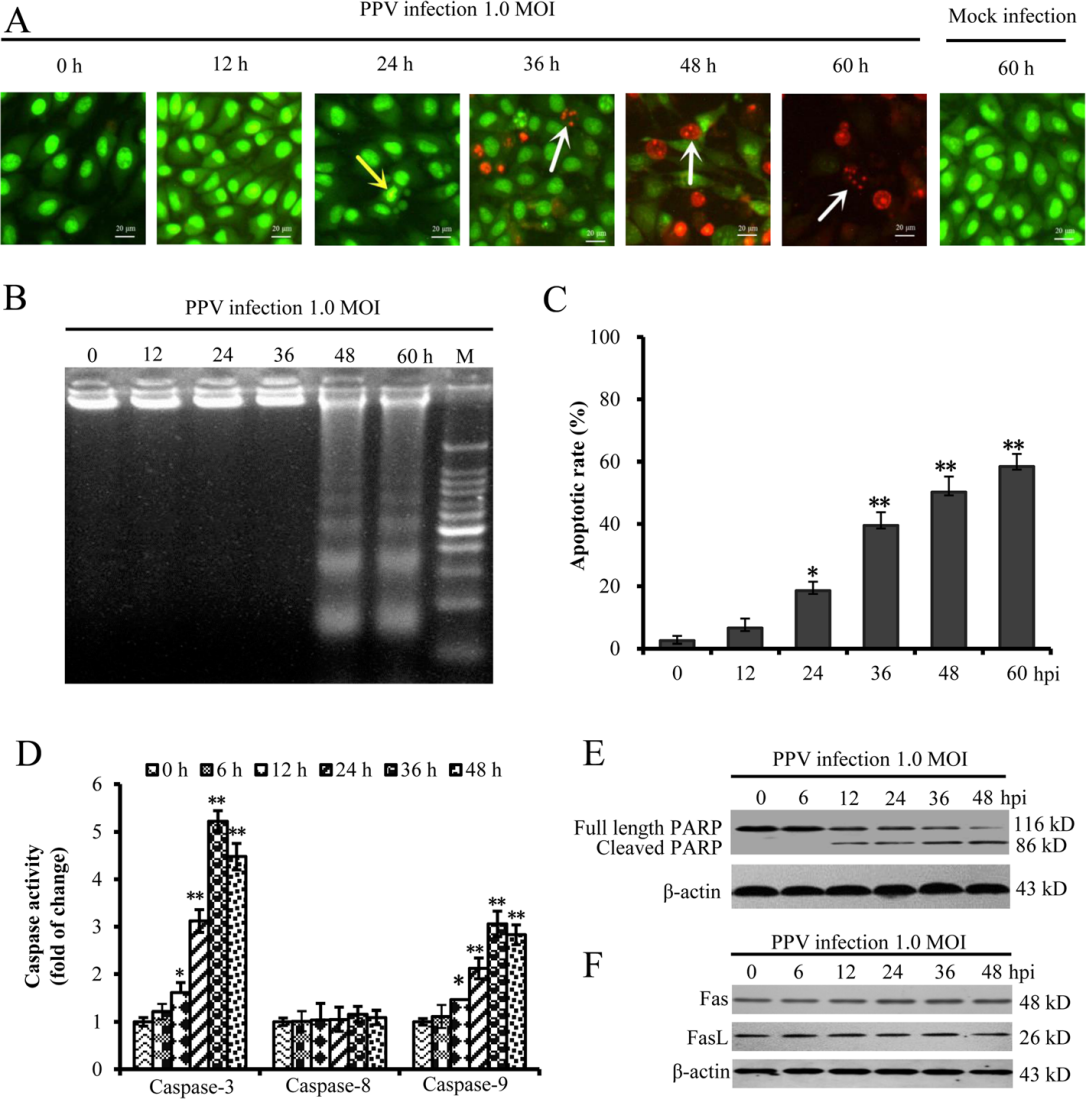
PPV infection induced ST cells apoptosis through activating caspase-9 and -3. **a** Morphology of PPV-infected ST cells. Morphology of PPV-infected cells was observed under fluorescence microscopy after AO/EB staining. The yellow arrow shows chromatin condensation, the white arrow shows nuclear fragmentation. **b** Analysis of DNA fragmentation in PPV-infected ST cells. **c** Analysis of ST cell apoptotic rate induced by PPV infection using flow cytometry. **d** The activity variations of caspases induced by PPV infection. The activities of caspase-3, -8 and -9 in PPV-infected ST cells at 1.0 MOI at 0, 6, 12, 24, 36, and 48 hpi were tested using the colorimetric assay kit. **e** Effect of PPV infection on PARP. The full long and cleaved PARP in PPV-infected ST cells were analyzed by western blot. **f** Effect of PPV infection on Fas and FasL. The presented data are the average of three independent experiments ± SD. *, *p* < 0.05; **, *p* < 0.01

To determine the role of PPV infection in apoptosis of ST cells, we observed the morphological change of PPV-infected ST cells. PPV-infected cells started to show typical apoptotic features including chromatin condensation (Fig. 1a, indicated by yellow arrows) after 24 hpi and nuclear fragmentation compared with mock-infected cells (Fig. 1a, indicated by white arrows) after 36 hpi. Next, we detected the pattern of chromosomal DNA fragmentation and found that DNA ladders appeared at 48 hpi in PPV infected cells at 1.0 MOI (Fig. 1b). Then the apoptotic rate was assessed using flow cytometry. As shown in Fig. 1c, the apoptotic rate of PPV-infected cells was increased at 24 hpi in comparison with the mock-infected cells and appeared time-dependent.

To investigate the mechanism of apoptosis induced by PPV infection, we further examined the activities of several vital caspases, including caspase-3, -8 and -9. The results indicated that the caspase-9 and -3 were activated after PPV infection at 1.0 MOI as early as 12 hpi (Fig. 1d). However the activity of caspase-8 was not affected by PPV infection. In addition, poly-ADP-ribose polymerase (PARP), an indicator of caspase-3 activation, was cleaved in PPV-infected ST cells at 12 hpi in a time-dependent manner (Fig. 1e). In addition, the Fas and FasL were not affected (Fig. 1f).

Taken together, these results indicated that PPV infection induced apoptosis in ST cells through activating caspase-9 and caspase-3.

### PPV infection activated the mitochondrial apoptotic pathway

**Fig. 2:**
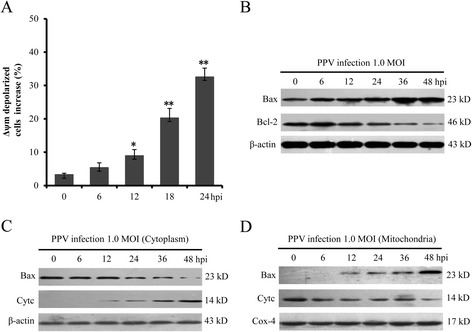
PPV infection activated apoptosis via mitochondria-mediated pathway. **a** The effect of PPV infection on of Δψm. **b** The expression level of Bax and Bcl-2 in PPV-infected ST cells. **c** Effects of PPV infection on Bax and cytochrome c in cytoplasm. **d **Effects of PPV infection on mitochondrial Bax and cytochrome c

Δψm plays an important role in the process of cell apoptosis. Once the Δψm is collapsed, mitochondria-mediated apoptotic signaling pathway will be activated. To determine the effect of PPV infection on Δψm, we detected the Δψm of PPV-infected and mock-infected ST cells. As shown in Fig. 2a, the percentage of Δψm-depolarized cells increased 6 % at 6 hpi and the level of depolarization was time-dependent. During mitochondria-mediated apoptosis, Δψm was regulated by Bcl-2 family members, such as pro-apoptotic protein Bax and anti-apoptotic protein Bcl-2. To explore the effects of PPV infection on Bcl-2 and Bax, western blot analysis was performed. The western blot results showed that PPV infection increased Bax expression and suppressed Bcl-2 expression (Fig. 2b).

To further detect whether the collapse of Δψm is associated with the release of cytochrome c and the translocation of Bax, the cytoplasmic and mitochondrial proteins were analyzed by western blot and respectively normalized to β-actin and Cox-4. The results showed that PPV-infection promoted the translocation of Bax from the cytosol to the mitochondria and enhanced the release of mitochondrial cytochrome c from mitochondria to cytosol at 12 hpi (Fig. 2c and d). All these results indicated that PPV infection activated the mitochondrial apoptotic pathway in ST cells.

### PPV infection induced ROS accumulation

**Fig. 3:**
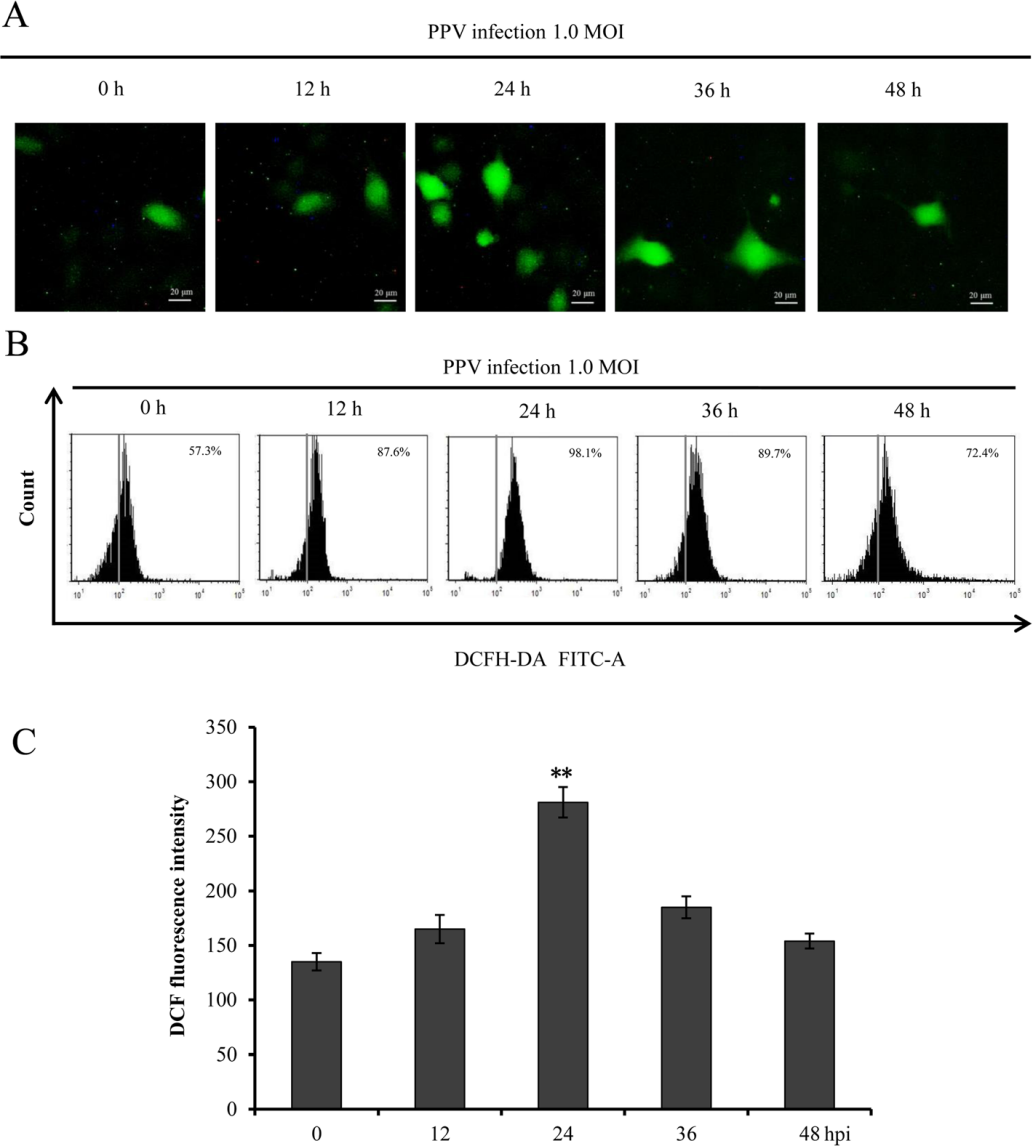
PPV infection led to ROS accumulation. **a** Observation of ROS production after PPV infection under fluorescence microscopy. Cells were infected with PPV at 1.0 MOI and harvested at 0, 12, 24, 36 and 48 hpi. Then the cells were stained with DCFH-DA and observed under fluorescence microscope. **b** Quantitative analysis of ROS in PPV-infected ST cells. The relative ROS level of cells infected with PPV at 1.0 MOI at 0, 12, 24, 36 and 48 hpi were analyzed by flow cytometry. **c** The levels of ROS for each infectious time were calculated by the fluorescence intensity in PPV-infected cells subtracting the fluorescence intensity in mock infected cells. The data are shown as mean ± SD of three independent experiments. *, *p* < 0.05; **, *p* < 0.01 in comparison with the control

ROS play an important role in the process of apoptosis [16, 17] and activate the mitochondria-mediated apoptotic pathway [19]. To investigate the role of ROS in the process of PPV-induced apoptosis, we detected ROS level in PPV-infected cells using DCFH-DA followed by observation under fluorescence microscopy. The results indicated that the fluorescence reached to the highest level at 24 hpi, and then decreased gradually along with the time course (Fig. 3a). To further demonstrate the ROS level, quantitative analysis of ROS in PPV-infected cells was performed using flow cytometry. As shown in Fig. 3b and c, ROS was significantly accumulated at 24 hpi in comparison with mock-infected cells and subsequently decreased at 36 hpi continuously with the extension of time. All the data indicated that PPV infection could trigger ROS accumulation in ST cells.

### The role of ROS in PPV-induced apoptosis

**Fig. 4:**
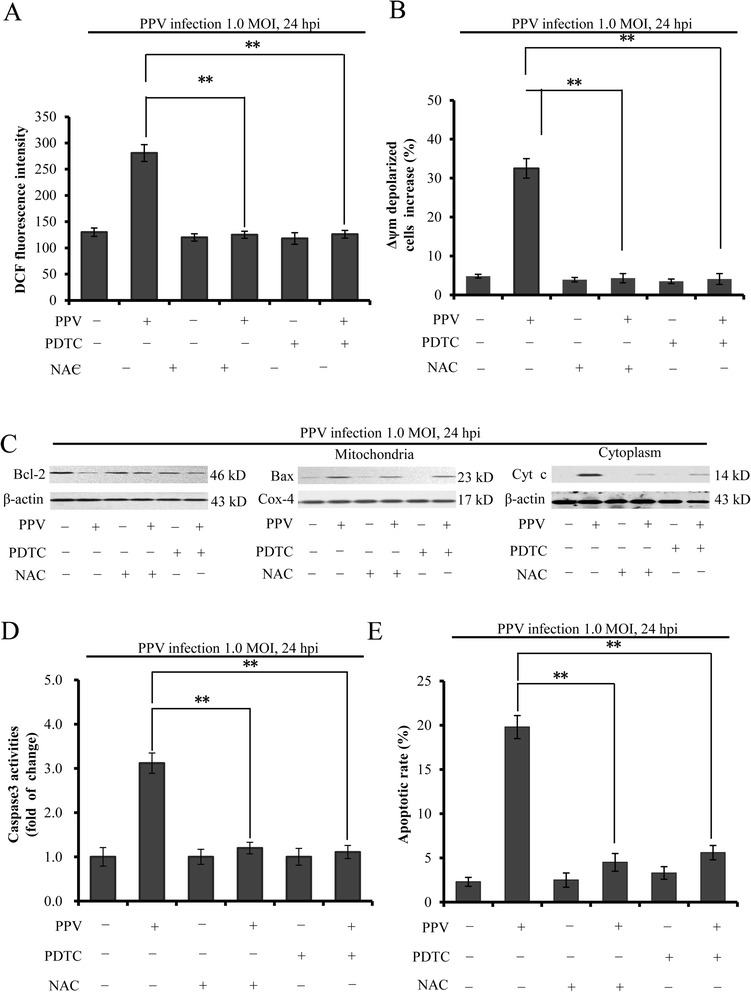
The role of ROS in PPV-induced apoptosis. **a** The effects of ROS inhibitors PDTC and NAC on ROS production. PPV-infected cells were treated with PDTC or NAC and stained with DCFH-DA. The ROS production was measured by flow cytometry. **b** The effect of ROS on Δψm in PPV-infected ST cells. The Δψm of cells infected with PPV and treated with PDTC or NAC was detected by flow cytometry. **c** The effects of ROS scavengers on the Bcl-2, Bax translocation, cytochrome c redistribution. ROS inhibitors-treated cells were infected with PPV and analyzed using western blot assay. The mitochondrial and the cytosolic proteins were respectively normalized to Cox-4 and β-actin. **d** The effect of ROS scavengers on the activity of caspase-3 in PPV-infected ST cells. Caspase-3 activity was measured using the caspase activity kit. **e** The effect of ROS scavengers on the apoptotic rate of PPV-infected cells. The apoptotic rate was measured by flow cytometry. The data represent three independent experiments ± SD. **, *p* < 0.01 in comparison with PPV infection alone

ROS accumulation may initiate cell apoptosis via activating the intrinsic mitochondrial pathway. Moreover, excessive ROS also leads to the damage of mitochondrial DNA and the permeabilization of mitochondrial outer membrane [20]. To further explore the effect of ROS on Δψm,we used ROS antioxidants PDTC (100 μM) (Merck, Germany) and NAC (100 μM) (Sigma–Aldrich, US) to suppress ROS production in ST cells infected with PPV at 1 MOI and 24 hpi and measured the ROS level and Δψm. Both PDTC and NAC, which did not show cytotoxicity at concentrations used (data not shown), significantly prevented the production of ROS (Fig. 4a) and reduced Δψm in PPV-infected cells (Fig. 4b). These results indicated that PPV infection decreased mitochondrial membrane potential through inducting ROS accumulation. To further investigate the role of ROS accumulation in apoptosis, we analyzed the expression of Bcl-2, translocation of Bax, and the redistribution of mitochondria cytochrome c in ST cells infected with PPV at 1 MOI, 24 hpi and treated with PDTC and NAC. As shown in Fig. 4c, the decrease of Bcl-2, the translocation of Bax from cytoplasm to mitochondria, and the release of mitochondrial cytochrome c to cytoplasm in PPV infected ST cells were blocked by PDTC and NAC treatment in comparison with these in PPV-infected cells alone. In addition, caspase-3 activity and apoptotic rate were remarkably decreased in the ST cells infected with PPV at 1 MOI and 24 hpi in the presence of PDTC or NAC (Fig. 4d and e). The data indicated that the accumulation of ROS induced by PPV infection contributed to the apoptosis at 1 MOI and 24 hpi.

## Discussion

Apoptosis plays a predominant role in contributing to the pathogenic process of virus infection [21] and presents specific morphological and molecular changes, including chromatin margination, DNA fragmentation [22, 23]. Previous studies showed that PPV infection induced PK-15 apoptosis via mitochondria-mediated pathway [19]. Evidence shows that oxidative stress is an important regulatory factor of virus-induced apoptosis and induced by various triggers, including viral infection [24]. In this study, we found that PPV infection induced apoptosis of ST cells via activating mitochondrial-mediated pathway and PPV-induced ROS accumulation was involved in regulating the process of apoptosis.

Apoptosis is primarily classified into two pathways regulating cell death involving the continuous activation of caspases. Of these, the extrinsic pathway participates in the ligation of particular “death” receptors (e.g., Fas) by recruitment of adaptor molecules to form the death-inducing signaling complex (DISC) and activate upstream caspases via caspase-8 [25]. The mitochondrion-mediated (intrinsic) pathway is characterized by changed mitochondrial membrane potential and release of cytochrome c from mitochondria into the cytoplasm. In the cytoplasm, cytochrome c then is involved in the formation of a multi-protein complex known as the “apoptosome” and activates the caspase cascade through caspase-9 [26]. In this study, we determined caspase-3 and caspase-9 were activated by PPV infection, but caspase-8 was not activated. This suggests that PPV infection induced the apoptosis of ST cells via mitochondrion-mediated pathway.

Previous studies indicated that Bcl-2 family proteins as major regulators, including Bcl-2 and Bax, regulated the integrity of mitochondrial membranes, and the destruction of the mitochondrial membrane potential leaded to the release of mitochondrial cytochrome c [27, 28]. Apoptosis can be triggered by the decreased mitochondrial permeability, which causes the release of apoptotic initiating factors, and the activation of caspase such as caspase-3. Some parvoviruses infection may lead to the increase of membrane permeability, subsequently inducing cell apoptosis. For example, bocavirus minute virus of canines (MVC) results in the time-dependent disruption of mitochondrial outer membrane potential (MOMP) [29]. Here we showed that mitochondrial membrane potential was significantly reduced in manner of time-dependent, which is consistent with the finding that MVC infection induced the disruption of MOMP.

The oxidative damage also leads to the release of cellular toxic proteins such as cytochrome c from the mitochondria to the cytosol, subsequently triggering apoptosis [14]. To explore whether ROS was involved in the apoptosis induced by PPV infection, we investigated the direct effect of PPV infection on the oxidative profile. Time course analysis of PPV infected ST cells showed that ROS accumulation increased significantly, suggesting that PPV infection induced ROS accumulation. In this study, two ROS inhibitors, PDTC and NAC, significantly attenuated PPV-induced Bax translocation, the release of cytochrome c, caspase-3 activity, and apoptotic rate. The data indicate that ROS accumulation induced by PPV infection increases mitochondrial outer membrane. Our finding is consistent with that the canine parvovirus (CPV) and rat parvovirus H-1 (H-1PV) nonstructureal NS1 protein induced apoptosis via accumulation of intracellular reactive oxygen species (ROS) [18, 30].

## Conclusions

Our data provide a link between ROS and apoptosis induced by PPV infection. The results demonstrated that PPV infection induced ST cells apoptosis via activating ROS accumulation and mitochondria-mediated apoptotic signaling. Our findings may provide insights into the interaction between PPV and host, and uncover the pathogenesis of PPV infection. Further studies on the mechanisms of PPV-induced apoptosis should give us new insights on the interplays between PPV and its host.

## Methods

### Cells, virus and antibodies

ST cells were purchased from American type culture collection (ATCC, CRL-1746) and cultured in DMEM (Gibco, US) culture medium supplemented with 10 % heat-inactivated fetal bovine serum (Gibco, US) at 37 °C in a 5 % CO_2_ humidified atmosphere. The PPV YL strain was used as previously described [19]. Virus titers were determined by 50 % tissue culture infective doses (TCID_50_) according to Reed and Muench method [31]. Mouse monoclonal antibodies against Bcl-2, Bax, Cytochrome c, Cox-4, PARP, Fas, FasL, and β-actin were purchased from Santa Cruz Biotechnology. Horseradish peroxidase (HRP)-conjugated secondary antibody was purchased from Promega Corporation.

### Effects of PPV infection on morphology of ST cells

Cells were seeded into 24-well plate and infected with PPV at 1.0 MOI. After incubated overnight at 37 °C, the cells were washed with PBS and incubated with 200 μl staining solution containing acridine orange (AO, 100 μg/ml) and ethidium bromide (EB, 100 μg/ml) at 0,12,24,36,48,60 hpi. After incubated for 10 min in the dark at room temperature, the cells were observed under a fluorescence microscope (Nikon, Japan). The normal cells can be stained by AO and show bright green fluorescence. The dead cells can be stained by EB and show orange fluorescence.

### DNA fragmentation analysis

ST cells were seeded into 100-mm dishes. When confluent to 50 % ~ 60 %, the cells were infected with PPV at 1.0 MOI and respectively harvested at 0,12,24,36,48,and 60 hpi. The cells were washed and lysed with 400 μl cell lysis buffer (20 mM EDTA, 100 mM Tris, pH 8.0, 0.8 % SDS) at room temperature for 1 h. Cellular DNA was extracted with phenol:chloroform:isopentyl alcohol (25:24:1), precipitated with ethanol, dissolved in TE buffer (10 mM Tris, pH 8.0, 1 mM EDTA), and analyzed with 2.0 % agarose gel electrophoresis.

### Caspases activities assay of caspase-3, -8, and -9

ST cells were infected with PPV at 1.0 MOI and incubated in a 5 % CO_2_ humidified incubator at 37 °C. The cells were harvested at 0, 6, 12, 24, 36, and 48 hpi, washed with ice-cold PBS for three times, and then lysed with 200 μl lysis buffer on ice for 1 h. The caspases activities were tested with colorimetric assay kit (Abcam, US) following the manufacture’s manual. Briefly, the protein concentration was measured using BCA Protein Assay Reagent (Pierce, US). 150 μg total protein of each sample was incubated with each caspase substrate in a 96-well plate for 4 h at 37 °C. The absorbance was measured at 400 nm with spectrophotometer (BioTek, US).

### Mitochondrial membrane potential (Δψm) assessment

The cells were harvested and stained with Tetrechloro-tetraethyl benzimidazol carbocyanine iodide 1 (JC-1) for 15 min at room temperature in the dark. Then the Δψm was assessed using JC-1 Mitochondrial Potential Detection Kit (Biotium Inc, US) following the manufacture’s protocol. The fluorescence signal was measured at 550 nm and 485 nm.

### Western blot analysis

ST cells were harvested and then lysed on ice for 1 h with radioimmunoprecipitation assay (RIPA) buffer with 1 mM phenylmethyl sulfonylfluoride (PMSF). Isolation of mitochondrial and cytosolic proteins was carried out using the Mitochondria/cytosol Fractionation Kit (Pierce, US) according to the manufacture’s protocol. Protein concentration was detected using BCA Protein Assay Reagent (Pierce, US). Equivalent amount of protein was loaded and subjected to electrophoresis on 12 % sodium dodecyl sulfate-polyacrylamide gel electrophoresis (SDS-PAGE). Subsequently, proteins were transferred to polyvinylidene difluoride (PVDF) membranes (Millipore Corp, US). The membranes were blocked with 5 % non-fat dry milk for 1 h at room temperature, and incubated with primary antibody at the dilution of 1:500 in antibody dilution buffer (PBS, 2 % non-fat dry milk) over night at 4 °C. The blots were subjected to secondary antibody at the dilution of 1:10000 in antibody dilution buffer (PBS, 2 % non-fat dry milk) at room temperature for 1 h and detected with ECL reagent (Pierce, US).

### The observation of ROS production under fluorescence microscope

The ROS were detected using Reactive Oxygen Species Assay Kit (Sigma–Aldrich, US). Briefly, ST cells were seeded into 24-well plate and infected with PPV at 1.0 MOI. Then the cells were stained with 10 μM 2’, 7’-dichlorodihydrofluorescein (DCFH-DA) at 0, 12, 24, 36, and 48 hpi and observed under the fluorescence microscope (AMG EVOS Inc, Israel).

### ROS analysis using flow cytometry

Mock-infected and PPV-infected cells were harvested at indicated times and washed twice with DMEM without fetal bovine serum. Then the cells were incubated with 10 μM DCFH-DA at 37 °C for 30 min in the dark and analyzed using flow cytometry (Beckman Coulter EPICS ALTRA, US).

### Statistical analysis

Statistical analyses were calculated using one way analysis of variance (ANOVA). Significance was attained at *p* < 0.05.

## Abbreviations

AO: Acridine orange
CPE: Cytopathic effect
EB: Ethidium bromide
hpi: hour post-infection
MOI: Multiplicity of infection
NAC: N-acetyl-L-cys-teine
PDTC: Ammonium pyrrolidinedithiocarbamate
PPV: Porcine parvovirus
ROS: Reactive oxygen species
ST: Swine testicular
TCID_50_: 50 % tissue culture infective doses
